# Anticoagulation and atrial fibrillation in prognostic models for type B aortic dissection

**DOI:** 10.1016/j.jnha.2025.100612

**Published:** 2025-06-16

**Authors:** Amir Reza Akbari, Benyamin Alam

**Affiliations:** a King's Mill Hospital, Sherwood Forest Hospitals NHS Foundation Trust, Nottinghamshire, United Kingdom; b Queen Elizabeth Hospital, Mindelsohn Way, Birmingham B15 2TH, United Kingdom

Dear Editor,

We read with interest the article by Zhao et al. titled “The role of geriatric nutritional risk index in predicting survival of type B aortic dissection patients after thoracic endovascular aortic repair” [1]. The study presents a meaningful analysis of the Geriatric Nutritional Risk Index (GNRI) as a prognostic marker for long-term outcomes in patients with type B aortic dissection (TBAD) undergoing TEVAR [1]. The findings underscore the importance of nutritional status in cardiovascular risk stratification, particularly in older populations.

However, we wish to highlight the omission of atrial fibrillation (AF) and oral anticoagulant (OAC) therapy from the study's baseline characteristics and multivariate analysis. This is a significant consideration, as both AF and its treatment are known to influence outcomes in TBAD [2]. A recent study examining the prognostic implications of AF and OAC therapy in patients with type B aortic dissection found that when AF patients were treated with OAC, the hazard ratio for MACCE increased to 3.875 (95% CI: 1.153–17.496) [2].

AF is a common comorbidity in the elderly, and its management introduces complex trade-offs in vascular pathologies, particularly where dissection and hemorrhagic risks intersect. Anticoagulation therapy may exacerbate bleeding or re-entry risk in dissection patients, which could in turn influence the outcomes attributed to nutritional status or procedural success. Given that the GNRI is also associated with systemic inflammation and cardiovascular frailty, failing to account for AF and OAC use could lead to confounding.

We encourage future studies on TBAD prognosis, especially those evaluating novel risk stratification tools like GNRI, to include atrial fibrillation status and anticoagulation therapy as covariates. This will provide a more comprehensive and accurate understanding of prognostic influences and support more individualized management strategies.

We commend the authors for their valuable work and hope this commentary contributes to further refinement of outcome prediction in this high-risk population.

## CRediT authorship contribution statement

All authors were equally involved in analyzing the original document and drafting the response. Each author participated in reviewing and refining the text and has read and approved the final version. The authors collectively take full responsibility for the content of the final document.

## Informed consent statement

Not applicable.

## Ethical approval

Not applicable.

## Funding

This research received no external funding.

## Data availability

Not applicable.

## Declaration of competing interest

The authors declare no conflict of interest.

## References

[1] Zhao K., Niu J., He Y., Kong L., Zhao W., Lu Q. (2025). The role of geriatric nutritional risk index in predicting survival of type B aortic dissection patients after thoracic endovascular aortic repair. J Nutr Health Aging.

[2] Ishizue N., Fukaya H., Oikawa J., Sato N., Ogiso S., Murayama Y. (2024). Prognostic impact of oral anticoagulation therapy and atrial fibrillation in patients with type B acute aortic dissection. J Arrhythm.

